# Extra Supplement—The Nursing Mirror

**Published:** 1890-06-14

**Authors:** 


Being the Extra Nursing Supplement of "The Hospital" Newspaper.

All Contributions for this Supplement should be addressed to the Editor, The Hospital, 140, Strand, London, W.C., and should have the word "Nursing" plainly written in left-hand top corner of the envelope.

### En Passant.

ONE OF THE ELITE.—In reference to the paragraph under the above heading which appeared in our issue of the 24th ult., we beg to state it was founded on the following letter from the registrar of the B.N.A.: "April 29th, 1890.—Dear Madam,—In reply to yours of the 28th inst., Miss Gertrude Johnstone has applied for registration, and I should be obliged, therefore, if you could give me any further information about her.—I am, dear Madam, faithfully yours, Helen Foggo Thomson, Registrar."

THE PRINCESS OF WALES AND THE FIRST THOUSAND.—H.R.H. the President of the National Pension Fund for Nurses has fixed Friday, July 4th, at one p.m., at Marlborough House, for the ceremony of presenting certificates to the first thousand nurses who joined the Pension Fund. The Princess of Wales has also graciously consented to receive the purses containing contributions to the Junius S. Morgan Memorial Benevolent Fund at the same time. All contributions must be sent to the secretary, National Pension Fund for Nurses, 8, King Street, E.C., on or before June 30th next. We are glad to hear that upwards of five hundred nurses have undertaken to obtain £5 each towards this memorial to the nurses' great benefactor.

UNIVERSITY COLLEGE HOSPITAL BAZAAR.—The Duchess of Fife very kindly opened this bazaar last week, though she was looking far from well. The nurses gathered in force, the blue gowns and spotted net caps looking very nice, and their stall was crowded with pretty articles, including some nurse-dolls. The Lady Pupils of Nursing had a stall for country produce, eggs, butter, and even fowls; Miss Cusins and Miss Wallace were the chief presiding spirits, and were dressed in the red uniform lately adopted. The weather was not very favourable, but the attendance was good, and the entertainments given by actors and musicians were much appreciated. The Duke of Fife spoke very nicely in replying to the address of Professor Erichsen, saying that the Duchess earnestly hoped the funds of University College Hospital would be materially increased by the bazaar which had been arranged at the expense of so much time and trouble.

INSOMNIA.—It has been our lot to chronicle once or twice the evil effects of morphia used by nurses; in one case the effect was fatal. Frequently a nurse loses all strength of mind or body by indulging in the habit of drug-taking, and we wish once more to most earnestly counsel all nurses never to take sleeping draughts save under medical direction. The habit is so easily formed, so hardly broken; and many a life is ruined thereby. A hospital nurse writing in one of the magazines lately, said: "One word on 'night duty,' to many nurses the most trying part of hospital routine, and I have done. Insomnia, the precursor of insanity, of mental lassitude, and bodily discomfort, is one of the most frightful evils to which flesh is prone. Owing to noise and daylight and kindred causes, nurses are peculiarly subject to it. But whether they wake or sleep throughout the day, twelve hours, sometimes longer, of 'night duty' remains to be accomplished. I venture to suggest that darkened rooms, remote from ordinary noise and bustle, and shorter hours (as in the Leeds Infirmary), would do something by way of remedy. Possibly under these circumstances we should hear less of morphia and chloral casualties. The necessary outlay would be more than counterbalanced by the mitigation of a very real and tangible calamity."

DISTRICT NURSING AT MANCHESTER.—A local paper lately had an account of a round of visits with one of the nurses of the Manchester Sick Poor and Private Nursing Institution. It is particularly in the houses of the decent poor that the labour of the nurses is most efficiently expended. The very poor are often so shiftless and unsatisfactory that care and nursing seems almost thrown away upon them; but with the honest and hard-working classes it is very different. A steady workman, with fair or moderate wages and a decent wife, can keep his head above water so long as his and his wife's health are good; but when illness attacks his home he is helpless. The poor are incapable of carrying out the doctor's directions to the extent of arranging the simplest poultice, bandage, or fomentation, and here the help of the trained nurse is invaluable. The account ought to arouse increased interest in the institution.

TO ASYLUM ATTENDANTS.—We give this week an introductory paper by Dr. Walmsley, of Leavesden Asylum, on the subject of "Asylum Attendants." This paper is to be followed by a series of articles entitled "Sketches of Insanity," which will concisely pass in review the salient features of insanity, and which should be most useful to those attendants who desire to progress in their work, but who are unhappily debarred from the teaching given to their more fortunate comrades. The subject of the training of the attendants in asylums, both in the rudiments of sick nursing and in the study of insanity, is one on which we feel very keenly. In every asylum a certificated hospital-trained nurse should be at the head of the infirmary ward, for the dying lunatic needs skilled attention as much as a sane man in a similar situation. Medical superintendents and matrons of asylums must see that the note of progress has been struck, and that attendants must now be chosen with greater care, and that systematic training must be given. The late scandals at Banstead Asylum show how necessary it is that a higher grade of women should be taken as attendants; we hope the medical superintendent there has learnt his lesson, and will in future pay more attention to the many subordinates over whom he rules.

A SUMMER SERMON.—Here we are, close to the longest day, and in the heart of the season of sunshine and song. "Ah!" says some reader, "what do these things matter to a hospital nurse?" They ought to matter a great deal more than they do, for the woman who loses the power of enjoying and idling through a long summer day, has lost an attribute without which a character is most imperfect. It is not the gospel of work, but the gospel of idleness which needs preaching to many of our nurses. Hospital life is so stimulating, so full of the suppressed excitement caused by constant contact with the great issues of life and death, that it absorbs a nurse's whole heart and mind, and takes from her the power to enjoy the simple pleasures of a summer day. The "man of one idea" is constantly held up to derision as a narrow-minded individual to be avoided at all costs; the hospital nurse is only too apt to become a woman of one idea. The way to escape this condemnation is to try for one day at least in every month to avoid all thought of patients, all talk about hospitals—to go away out into the sunshine and the green fields, to lie beneath the trees, and open the heart to the sweet, hopeful teaching of Nature in her summer garb. Life is short, and full of labour and anxiety for most of us, and we will never comprehend it aright unless we pause at times and turn aside from the city's roar to listen to the story of the birds in the woods. Let all try to obtain a few days of rest and peace before the summer loses its present beauty.

### Asylum Attendants.—Their Work and Training.

By Francis H. Walmsley, M.D.

Quite recently a committee of the London County Council reported that a lunatic hospital would be likely materially to increase the present knowledge of the nature and causes of insanity, and therefore ultimately to increase the means available for its prevention and cure. There can be little doubt that the asylum of the future will approximate more and more in character to the hospital type, and that we shall at no distant date see such hospitals established, not only in London, but also in the large towns of Great Britain.

There prevails an uneasy feeling that the places in which the insane are received are too much asylums and too little hospitals.

The progress of knowledge more and more indicates that insanity is simply a disease subject to the same physical conditions as other diseases, that the brain function—mentalization—is disordered, and that insane patients should ever be regarded as *sick* men and women, calling for the same active treatment and close watching as is given to a case of typhoid fever or diphtheria.

The need of a superior and permanent staff as an element in the treatment of cases of lunacy is one of the most patent necessities in asylums, and the future training of attendants is certain to undergo an improvement as remarkable as the changes which have occurred in the treatment of the insane.

Attendants are necessarily the instruments by which all the details of treatment are brought into practice, yet those who take to the service are generally persons devoid of all training.

We strongly think that in the interests of all concerned it is most desirable that special and systematic training should be given to asylum attendants, and it is satisfactory to find that already many asylums have given practical effect to this principle.

As a result of the training which all hospital nurses have now to undergo, we see how materially their "status" has been improved; we see that they are universally regarded as the right arm of the medical service; and we would urge the importance of extending to all asylum attendants opportunities and facilities whereby they may be enabled to qualify themselves satisfactorily to perform the higher and more specialised functions they will be called upon to undertake.

The cardinal principle of all treatment of the insane is—by bringing into operation every diversity and variety of influence—to prevent the formation of a morbid habit of thought, and the prompt arrest of this brain habit where already formed. This cardinal principle the asylum attendant is called upon to reduce into practice; surely a difficult task to set one to whom there has never been explained the nature or the mode of onset of a delusion or hallucination. The above considerations alone would suffice to demonstrate that thorough training and special education are essential for every asylum attendant.

It is pleasant to note that governing bodies are coming into closer touch and sympathy with their officials of all grades. They fully recognize that—perhaps to a greater extent than any other influence—the personal qualities of every member of the medical and nursing staff (as one of the Commissioners in Lunacy happily expressed it) is really the *cure*. They fully realize that the duties of the attendants are arduous and exhausting, equally do they recognize that if these duties are to be faithfully and efficiently performed, means must be found whereby those having charge of the insane may be enabled to preserve a vivacious mind in a vigorous body. We see this concern for the comfort and well-being of the staff daily displayed; they are provided with comfortable quarters, with recreation rooms, with good and varied dietary, with more liberal extension of leave, and with means of healthy amusement and occupation. Most asylums are now provided with tennis courts in some distant part of the grounds, so that the nurses may—when off duty—get right away from both wards and patients.

In every walk of life do we see that the individual whose well-conserved life shows itself in overflowing spirits becomes by his mere presence a source of pleasure to all around. Such are the individuals best fitted to become asylum attendants.

### Our Bird Cage.

Dear Sir,—Of course every nurse considers that her hospital is better than any other, and when asked for a reason why this strange belief there is always a something which her hospital possesses which no other does. Thus it is that ours is certainly unique, and for why? It once possessed a doll's house, which is now a bird cage, and the conversion took place this way: One of our number has a guardian. We don't feel jealous, and we don't want guardians ourselves. We are quite willing that "the Ward" (she is called "The Ward" to distinguish her from the natural wards of the hospital) shall have her guardian all to herself, but such a thing has never occurred within the recollection of the establishment, and, therefore, naturally we want to know something more about the guardian, for when the ward wants anything she simply says her "guardian will do it," and he does. Every morning a small triangular packet arrives (we all feel sorry for the small porter, he has a deal to carry to that ward), and the contents a tiny bouquet of flowers. A few have been privileged enough to see the guardian, but we poor outsiders, and there are some thirty of us, have only a faint idea of what he is like by peeping into the ward's room, for there is his likeness, not an aged man of 70—as we expected, a veritable stern father of a family, but forsooth against all precedent a confirmed old bachelor (we think the ward considers him young, but we don't), sitting in an arm-chair, reading and smoking. We also saw fretwork brackets. The guardian's manufacture! We wonder how the ward keeps them clean; we shouldn't like the trouble. The ward had a holiday. The guardian, we heard, came all the way to fetch her, and when she reappeared she brought two canaries. This was the cause. The head nurse suggested that the doll's-house would look very pretty as a bird-cage. The ward tied a piece of string round it, as being the simplest mode of packing, and sent it to the guardian. What the guardian thought on the receipt of a large doll's house, six windows in a row, and a staring, green balcony, relieved by red brick walls, we can't imagine, and what he thought of the ward's request we can't even conjecture; but one thing we do know, it went a doll's-house and came back a bird-cage. The rooms are gone, all that remains is the front. The glaring balcony, the pride of former days, is replaced by a covered verandah, all wired in, the windows being left open spaces. The two windows each side of the front door are likewise done, and make seed boxes. The front door, a staring symphony in green and red, is replaced by folding doors of oak, with open windows. What was the house itself is now the cage, fitted entirely with twigs and a merry-go-round of the same materials. There are two nests and nine doors.

It returned in an open crate and wherever it sojourned received a small admiring audience.

The guardian has evidently a sense of humour and it was a picture seeing the ward viewing that cage. Lucky for the guardian he was absent (for we don't consider the ward is the meek little thing she looks, or why is the guardian so obedient?). Our hospital is of course the county one, so the cage has three large and elaborate designed letters over the verandah—C. C. H., cut out in fretwork, County Canary Hospital. Then the notice boards—it was these the ward was reading first of course. "Under the distinguished patronage" of our matron, followed by an urgent application for funds. There was a vacancy for a probationer, small premium required, likewise the inmates desired a chaplain to be chosen by the nurses, a request that the doors were not to be opened as the patients might get out. At first we thought these notices were satire, but there was one that convinced us we were wrong. The doctors are requested to open the ward doors for themselves, and study politeness. Whoever heard the doctors requiring the nurses to open the door for them or using other than polite language, and how on earth could it get to the guardian's ears? Did the ward tell him?—Yours truly,

One of the Insiders.

P.S.—I forgot to tell you the ward was seen to go into the photographer's in full costume; perhaps the guardian admires it, we think it hideous, and the cloaks are really dangerous to wear. We can't move in them; just like sacks, only worse.

### Everybody's Opinion.

*[Correspondence on all subjects is invited, but we cannot in any way be responsible for the opinions expressed by our correspondents. No communications can be entertained if the name and address of the correspondent is not given, or unless one side of the paper only be written on.]*

### THE REGISTRATION OF MIDWIVES.

Mrs. Nichols, Secretary of the Midwives Institute, writes: As the question of the registration of midwives is now creating a certain amount of discussion, I venture to send you the following statement. The object of the Bill is to protect mothers from unqualified midwives, a reform which is urgently needed for the safety of working women, among whom it is calculated that out of every ten cases of childbirth seven take place without the assistance of any medical man. The lives of mothers are daily sacrificed by the action of ignorant persons who can at present undertake the duties of a midwife without let or hindrance, although this calling requires careful training and guarantees of efficiency. The Bill which Mr. Henry Fell Pease, M.P., introduced this session, and which has been promoted by the Midwives' Institute, will only affect those women who use the title of midwife, and thus proclaim themselves competent to fulfil the duties entailed. Care will be taken not to prevent neighbourly offices, whilst both the public and the qualified midwife will be protected against the ignorant and untrained women who at present prey on both alike. The interests of those midwives already in practice will receive full consideration. In the interests of the public, Parliament in 1878 and in 1883 passed analogous measures, which provided that no one should assume the title of dentist or of veterinary surgeon without holding a diploma of efficiency. But no protection has yet been accorded to English mothers who are without any assured means of ascertaining that the midwives (so called) whom they employ are in any way qualified for the responsible duties they undertake. In view of the urgency of the case, we therefore earnestly ask for the support of the public, so that the Bill may be carried through this Session, and may give that protection to women in our own land which they can command in every other European country.

### A HOME OF REST.

Mr. Josiah Oldfield, M.A., writes:—I think your note is expressive of a distinct truth—nurses for a change do not want merely that variation which consists in transferring from institution life with work to institution life without work; but a radical variation in the whole environment, which can, perhaps, best be obtained by joining a country family, even though it be but a cottage family, with all the freedom of restful surroundings; without responsibility, without worry, without restricting regulations, without any temptations to keep the mind towards its old groove. I feel sure that such could be obtained for nurses, to whom all owe so much, for about 12s. per week. I think I could find in the western counties (of which I know more of the village life) one or two congenial homes where the primitive simplicity of farmhouse-life is accompanied by such mental culture as would make them most agreeable for resting places for a thorough summer change. I shall be pleased to be of any assistance to anyone who is wishful to find such a change.

"Matron" writes: Will you allow me to add my opinion to those already expressed that what nurses require more than a home of rest is longer holidays. A fortnight once a year is not enough for a woman whose work is so hard and continual as that of a hospital nurse. Three weeks should be the minimum allowance of all nurses, and I prefer the nurses to take this in two instalments, a fortnight being taken for the annual summer holiday, and a week being kept in reserve for a change which may be wanted later on. No nurse should be expected to work from one summer holiday to another without a break. That most hospital nurses do exist on a lamentably short allowance I am well aware, and many of them are able to keep on under this continual strain, but the statement I read lately is nevertheless only too true, that a more anæmic-looking set of women than hospital nurses it would be difficult to find. It does not seem to me that during the holidays rest is not so much needed as a complete change of occupation. Active pursuits, such as boating, tennis, cycling, and riding, from which the nature of their profession shuts nurses out at all times except during their too brief holiday, are more keenly enjoyed, and serve as a means of relaxation and recreation more than any more passive form of occupation could do.

### "THE ANÆMIC LOOK."

Miss A. E. Wood, matron of the Royal Infirmary, Glasgow, writes: "In The Hospital of May 24th, attention is called to the anæmic look of nurses met by chance in the street. Reading further, I find that private nurses are more especially referred to. Of that side of the question I have but limited personal knowledge, and that many years old. I believe it is a fact that there is a considerable amount of anæmia among nurses, and I am convinced that one cause is, that many hospitals accept as probationers girls of twenty and even younger. Though apparently in the most robust health at starting, they have not come to their full strength either mentally or physically; they go on well for a time, pluck and spirit helping them, then gradual failure, and just when their training is beginning to bear fruit. Of course, too, the thoughtlessness, if not worse, of some employers contributes to some extent. Since we rigidly refused to admit as probationer any person who had not attained the age of 24, the health of the nurses has manifestly improved, and now anæmia is becoming a disease of the past.

### THE JUNIUS S. MORGAN MEMORIAL.

J. F. F. writes:—I sincerely hope that the "Junius S. Morgan Fund" is to extend its benefits to "Mental Nurses" as well as Hospital trained. Surely they are equally deserving, being often brave women, using nerve, tact, thought, watchfulness, and great self-control. Why do they get no word of praise or help? Surely the "fund" must include the one as the other, in its benefits to old age.

### ASYLUM ATTENDANTS.

"Only a Mental Nurse" writes: I also read with pleasure the letter in The Hospital for April 26th, written by "P. B.," referring to the status of asylum nurses, and also an asylum matron's. My sister and I trained at Hanwell and Banstead Asylums, though now private nursing. My father was in fair circumstances, we had a good plain education, and a dear good mother who brought us up carefully; we need not have left home, but we girls wished to be independent. I now belong to a large institute, and mix a good deal with hospital nurses, and, I say it in all humility, I feel I am quite equal, or even superior, intellectually to some of the hospital-trained nurses.

### Examination Questions.

Twenty answers were received to the May question, the best being that of Nurse E. M. Boutell, to whom we have sent "Questions and Answers on Nursing," by J. W. Martin, M.D. Two very good answers were received from Scotland—one from Coldstream and the other from Cupar. A somewhat lengthy communication from St. Leonards wandered from the subject, and though we were very much obliged to those nurses who told us how to treat diphtheria from a medical point of view, we did not dare to consider their papers, our own information being solely that of a nurse. As usual, a good paper came from Oxford, but in her enthusiasm for her patient, the writer had forgotten to mention the precautions for the nurse's own health. On the whole, Nurse Boutell's answer, which we will print next week, was the best, though it makes no mention of the respirator a nurse is often bidden to wear while attending to a diphtheria patient.

### A NURSE'S WORK.

"Let me ask you, nurses, whether this position is not a sufficiently lofty one, a sufficiently responsible one to satisfy the ambition of any woman. She whose interests are placed in your keeping during the absence of the physician, a time ordinarily almost equal to twenty-four hours out of the day, is, let us suppose, the mother of a family in the higher walks of life. She is some man's social idol, the head of a household of growing children, a leader in the society which her presence adorns, an originator of and a worker in many of the numerous beneficent enterprises which make our century so notable; a pivot around which revolve usefulness, charity, kindness, and love. Her physician leaves her at a most critical period of her existence, perhaps after the performance of a desperate operation. To your intelligent care, your strict watchfulness, your trained senses, are entrusted these interests, greater far to the loving circle whose eyes and hearts and prayers are upon your patient, than any others which the world contains."—*Dr. Gaillard Thomas.*

### Notes and Queries.

To Correspondents.—1. Questions may be written on post-cards. 2. Advertisements in disguise are inadmissible. 3. In answering a query please quote the number. 4. If a private answer is desired, a stamped addressed envelope must be forwarded.

Notice.—We must really ask our correspondents to be more particular in enclosing name and address. Constantly we receive queries or letters with no name attached.

### Queries.

(19) *Canary Isles.*—Two nurses will be glad of any information as to nursing in these Islands.

### Answers.

*Holland Institution.*—The directress of the Hollond Institution, Nice, thanks all the nurses who have written to her, but can only answer personally some of the most suitable.

*J. F. F.*—We do not undertake to insert all letters sent us; space forbids our printing more than the most important.

*Nurse A.*—"A Manual of Testing," K. M. Heanley, price 1s. 6d., from H. K. Lewis, 136, Gower Street, W.C. We will carry on the examination questions again next autumn.

*Nurse W.*—We cannot supply you with the addresses of American doctors. The P. and O. do not take trained nurses as stewardesses; the Orient line has done so sometimes.

*F. D.*—Your enclosure has been forwarded to (15.)—*E. H.*

*M. D.*—Why have you crossed out your name and address? We should like to publish your letter but you must send name and address—not necessarily for publication, but as a guarantee of good faith.

*Wandsworth Nurse.*—Certainly you must not send some one else to Marlborough House if prevented from going yourself. Such a course would be likely to bring both the substitute and yourself into serious trouble, besides being most disrespectful to the Princess.

*L. E.*—Get a bonnet and cloak, they will always be useful. Our correspondence is so great we cannot reply privately except when it is absolutely necessary.

*L. P.*—We can only advise you to read "The Joys of Johannesberg," in last week's number, and to stay at home.

### Vacancies.

The following vacancies are announced:

### Night Superintendent.

Worcester General Infirmary, £27.

### Nurses.

Burton-on-Trent Institution; £25.

Berry Wood Asylum; £20.

Blackheath Nursing Institution.

Blackburn Infirmary; £25.

Bishop's Stortford Institution; £25.

Birkenhead Borough Hospital; £25.

Brixton Institute.

Clayton Hospital, Wakefield; £25.

Canterbury Nurses' Institution.

Clapham Institution.

Croydon Institution; 35.

Chelsea Infirmary; £18.

Deaconess' House, Chester; £24.

General Nursing Institute.

Hanwell Ophthalmic School; £26.

Highbury Nurses' Institution.

Hospital, Victoria Docks.

Huddersfield Nurses' Home.

Jersey Nurses' Institute; £25.

Levick Institution, Paris.

Liverpool Institution.

Lock Hospital, Harrow Road.

Metropolitan Convalescent Institution; £20.

Montreal, Canada; £37.

North-West London Hospital; £24.

New Hospital for Women, N.W.

North Surrey District School; £25.

North London Consumption Hospital; £23.

Nurses' Home and Institution, Sheffield.

Nurses' Institute, Weston-super-Mare.

Oldham Workhouse; £30.

Queen Victoria Nurses' Institute (Scottish Branch).

R.S.C. Hospital, Guildford; £16.

Shoreditch Infirmary (night); £20.

St. Helena Home.

Surbiton Institution.

South-Eastern Fever Hospital; £30.

St. Cuthbert's Poorhouse Hospital, Edinburgh.

Swansea and South Wales Nursing Institution; £25.

Swansea General and Eye Hospital; £25.

Western Fever Hospital; £30.

Walsall Cottage Hospital; £18.

West Ham Hospital; £24.

Workhouse Infirmary Nursing Association.

### District Nurses.

East London Nursing Society.
Hayes, Kent; £52.
Jersey Nurses' Institute.
Middlesboro' Nurses' Home; £25.
St. Andrew's, Plymouth.

### Probationers.

Bedford General Infirmary.

Bristol Hospital for Sick Children and Women.

Clapham Institution.

Clayton Hospital, Wakefield; £12.

Grimsby Hospital.

Highgate Convalescent Hospital.

Hospital for Sick Children, Gateshead-on-Tyne.

Queen Victoria Nurses' Institute (Scottish Branch).

Royal Hospital for Diseases of the Chest; £12.

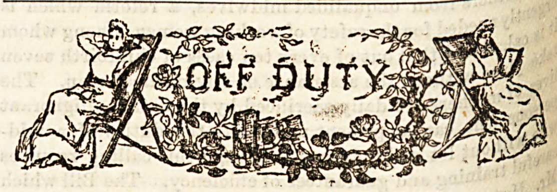

### Amusements and Relaxation.

**N.B.—New Word Competition Commenced April 5th, 1890, ends June 28th, 1890.**

Proper names, abbreviations, foreign words, words of less than four letters, and repetitions are barred; plurals, and past and present participles of verbs, are allowed. Nuttall's *Standard* dictionary only to be used.

The word for dissection for this, the ELEVENTH week of the quarter, being

"CHERRIES."

| Names. | June 5th. | Totals. | Names. | June 5th. | Totals. |
|---|---|---|---|---|---|
| Adeline | — | 149 | Agamemnon | 44 | 396 |
| Patience | 40 | 393 | G. E. J. C. | — | 34 |
| Lightowlers | — | — | S. E. A. | — | 24 |
| Canary | — | 86 | Jenny Wren | 35 | 362 |
| M. E. S. | — | — | Multum in parvo | — | 32 |
| Ambition | — | — | Ecila | — | 39 |
| M. W. | — | 217 | Weta | — | 259 |
| Esperance | — | 238 | Henri | — | 161 |
| Judy | — | 37 | Holland | 41 | 392 |
| Reldas | 42 | 393 | T. J. | — | 111 |
| Merenda | — | 29 | Nurse Isabel | — | 17 |
| Lucy Locket | — | 40 | Nurse Faith | — | 9 |
| Camellia | — | 30 | E. S. Z. | — | 10 |
| Qu'appelle | 37 | 376 | G. P. | — | 10 |
| Tinie | 43 | 396 | Stumps | — | 33 |
| Nurse Mildred | — | 190 | Bolton | — | 8 |
| Coralie | 39 | 388 | Jesmur | — | 53 |
| Gladys | 38 | 386 | Ran | — | 17 |

### Notice to Correspondents.

N.B.—Each paper must be signed by the author with his or her real name and address. A *nom de plume* may be added if the writer does not desire to be referred to by us by his real name. In the case of all prize-winners, however, the real name and address will be published.

Competitors can enter for *all* quarterly competitions, but no competitor may take more than *one first prize* during the year.

*** Correspondents whose communications do not appear in the current number are requested to refer to the following week's issue.

